# Disparities in Diabetes Technology Uptake in Youth and Young Adults With Type 1 Diabetes: A Global Perspective

**DOI:** 10.1210/jendso/bvae210

**Published:** 2024-11-28

**Authors:** Rebecca Baqiyyah Conway, Janet Snell-Bergeon, Kyoko Honda-Kohmo, Anil Kumar Peddi, Salbiah Binti Isa, Shakira Sulong, Laurien Sibomana, Andrea Gerard Gonzalez, Jooyoun Song, Kate Elizabeth Lomax, Ching-Nien Lo, Wondong Kim, Aveni Haynes, Martin de Bock, Marie-Anne Burckhardt, Savannah Schwab, Kwanho Hong

**Affiliations:** Department of Epidemiology, University of Colorado, Aurora, CO 80045, USA; American Academy of Epidemiology, Inc., Tyler, TX 75701, USA; Department of Pediatrics, Barbara Davis Center for Diabetes, University of Colorado, Aurora, CO 80045, USA; Division of Preventative Healthcare, National Cerebral and Cardiovascular Center, Suita, Osaka 564-8565, Japan; Technical Head, Cgenic Meditech Pvt Ltd, Gujarat 390020, India; Universiti Sains Malaysia, Advanced Medical and Dental Institute, 130200 Pulau, Pinang, Malaysia; Division of Medical Operations, Metro Sihat Sdn Bhd, 60000 Kuala Lumpur, Malaysia; Department of the Director, Pillar of Health, Pittsburgh, PA 15237, USA; Department of Pediatrics, Barbara Davis Center for Diabetes, University of Colorado, Aurora, CO 80045, USA; Department of Psychiatry, Jooyoun's Psychiatry, 07938 Seoul, Korea; Department of Endocrinology and Diabetes, Perth Children's Hospital, Nedlands, WA 6909, Australia; Children's Diabetes Centre, Telethon Kids Institute, The University of Western Australia, Nedlands, WA 6909, Australia; GM Office, EPS BIO Technology Corp., Hsinchu 30076, Taiwan; Management (Including R&D Director), CareforU Co., Ltd., 14042 Anyang, Korea; Children's Diabetes Centre, Telethon Kids Institute, The University of Western Australia, Nedlands, WA 6909, Australia; Department of Pediatrics, University of Otago, Christchurch 8140, New Zealand; University Children's Hospital Basel UKBB, Pediatric Endocrinology and Diabetology, 4056 Basel, Switzerland; Department of Epidemiology, University of Colorado, Aurora, CO 80045, USA; Management (Marketing & Development), CareforU Co., Ltd., 14042 Anyang, Korea

**Keywords:** type 1 diabetes, disparities, diabetes technology, continuous glucose monitor, continuous subcutaneous insulin infusion, automated insulin delivery system

## Abstract

Globally, nearly 9 million people are living with type 1 diabetes (T1D). Although the incidence of T1D is not affected by socioeconomic status, the development of complications and limited access to modern therapy is overrepresented in vulnerable populations. Diabetes technology, specifically continuous glucose monitoring and automated insulin delivery systems, are considered the gold standard for management of T1D, yet access to these technologies varies widely across countries and regions, and varies widely even within high-income countries. This review focuses on disparities in diabetes technology use among adolescents and young adults with T1D, barriers to access and use, and summarizes common themes emerging across countries and regions. We conducted a survey among medical technology manufacturers and physicians in various countries across diverse geographical regions and performed extensive literature searches. Across all countries and regions, economic barriers stand out as the largest and most common barriers, either preventing market penetrance of technology into a country or limiting its access to the individual with diabetes due to high out of pocket costs. Other common barriers include structural or accessibility barriers, such as stringent eligibility requirements by insurance providers, regardless of whether the insurance was private or government-based, and provider/individual level barriers. Based on the evidence presented, we suggest the need for a joint effort involving governments, private health insurers, technology manufacturers, and healthcare providers to address the global disparities of diabetic technology utilization and ensure equitable access for all individuals living with T1D worldwide.

Worldwide, nearly 9 million people are living with type 1 diabetes (T1D), an autoimmune disease that requires lifelong treatment with exogenous insulin. India, Brazil, and China account for the largest number of people with T1D, while incidence and prevalence rates are highest in Kuwait, Saudi Arabia, and Finland [1]. Although the burden of T1D is spread across high-, middle-, and low-income countries, the ability to treat, manage, and survive with the disease shows vast disparities, both between and within countries, resulting in wide global variation of survival rates and years of disability free quality of life. A cornerstone to slowing down the progression of complications of diabetes and their resulting disabilities and early mortality is optimizing glycemic management.

Over the past several decades, tremendous advances in diabetes therapies and technologies such as insulin and insulin analogs [2, 3], continuous glucose monitors (CGM) [4], point of care testing of blood glucose, and HbA1c, in-home blood glucose monitors, continuous subcutaneous insulin infusion (CSII), or insulin pumps [5, 6], sensor augmented pump (SAP), and automated insulin delivery (AID) systems [7-9] have led to improvements in glycemic control and have reduced the incidence of severe hypoglycemia, diabetic ketoacidosis (DKA), and longer term diabetes-related complications. Diabetes technology, specifically CGM and AID, are considered gold standard for management of T1D, yet access to these technologies varies greatly both between countries and within some high-income countries.

This review describes disparities in diabetes technology use among adolescents and young adults with T1D across and within countries and regions, barriers to access and use, and summarizes common themes identified across countries and regions. We additionally aim to present the impact of disparities in technology use by describing the incidence and or prevalence of T1D in selected countries, the market penetrance of diabetes technology in that country, and where data are available, variability in that penetrance, and the years of life lost (YLL) due to T1D in that country. This report represents a joint effort between diabetes epidemiologists, physician scientists in the fields of endocrinology and psychiatry, laboratory-based clinicians, and diabetes medical products manufacturers.

## Methods

**Figure 1.**
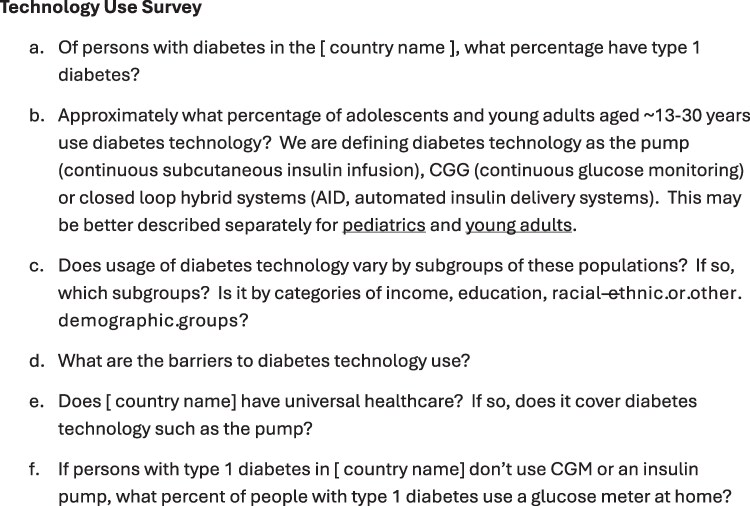
The survey used by the coauthors representing the countries in this mini-review in gathering the information presented in this mini-review.

A survey was conducted of medical technology manufacturers and physicians in various countries in conjunction with a literature search to determine advanced technology use including disparities in and barriers to optimal uptake (Fig. 1). The purpose of the surveys was to facilitate the gathering of data on technology use, disparities in use, and barriers to use. Although for some high-income countries, particularly high-income Western countries, the scientific research on technology use, disparities in technology use, and barriers to technology use in T1D are fairly well developed, for many countries (and where the majority of persons with T1D live), this published literature does not exist. Thus, the surveys were a combination of original research as well as an aid to help the literature search. The authors representing the various countries were asked to gather for their country the information requested in the survey. Where these data were not formally published, as is the case for many countries around the world, the authors asked T1D stakeholders (treating physicians, T1D patient groups, etc.) or searched national databases on Ministries of Health. Eighteen surveys were completed, with some surveys covering multiple countries (specifically, 1 survey covered only Switzerland and another survey covered the remaining European countries presented in this review). The specific countries included in the survey were Canada, the United States, Mexico, Brazil, Japan, Korea, Taiwan, India, Malaysia, Australia, New Zealand, Kenya, Rwanda, South Africa, Germany, Austria, Luxembourg and Switzerland, Denmark, Sweden, and Norway. This was a convenience sample of selected countries on each continent, with the countries selected based on the existence of published data in the literature, and the countries in which the coauthors resided or had access to information.

Databases searched and sources of data included PubMed, Japan's Medical Literature Search Service-Medical Online (medicalonline.jp), country-specific Ministries of Health (India, Korea, Malaysia), country-specific department of public health databases, the India Medical Association; stakeholder groups such as endocrinologists, diabetes patient groups, diabetes associations including the Japan Society of Pediatric Insulin, the Yilan County Love Pancreas Association in Taiwan, the Children Diabetes Community in Korea, and market research by diabetes technology manufacturing companies with a wide sales network, sales force, and distributors throughout the specific country. Search terms for literature database searches included type 1 diabetes, type 1 diabetes_cohort study, CSII_problems, diabetes technology, CSII, pump, closed loop hybrid system, automated insulin delivery system, CGM, continuous glucose, monitoring, disparities in diabetes technology use, diabetes technology use in [country], diabetes technology use in Africa, diabetes technology use in Latin America, CGM in Latin America, CGM in [country], barriers to diabetes technology use, barriers to diabetes technology use in [country], diabetes management in X or Y country, continuous glucose monitor in X or Y country, insulin pump use in X or Y country, diabetes outcomes in [country], diabetes complications in [country], cost of type 1 diabetes in [country], as well as the same questions for Latin America or Africa as a group.

### Key terms

The diabetes technologies focused on in this review are CGMs, CSII, AID, and SAP. Although the term youth and young adults varies in how it is defined both across and within countries, depending on the context, the clinic, research study or health organization reporting the data, in this manuscript we are generally referring to individuals younger than age 30 years. YLL is a measure of premature mortality that takes into account the average life expectance of the general population and the average age of death from a specific disease. It is the difference in the average age of death in a specific country's general population and the average age of death from a specific disease in that country, in this review being T1D. YLL presented in this review was obtained from the Type 1 Diabetes Index [1].

## Disparities in Diabetes Technology Use

**Figure 2.**
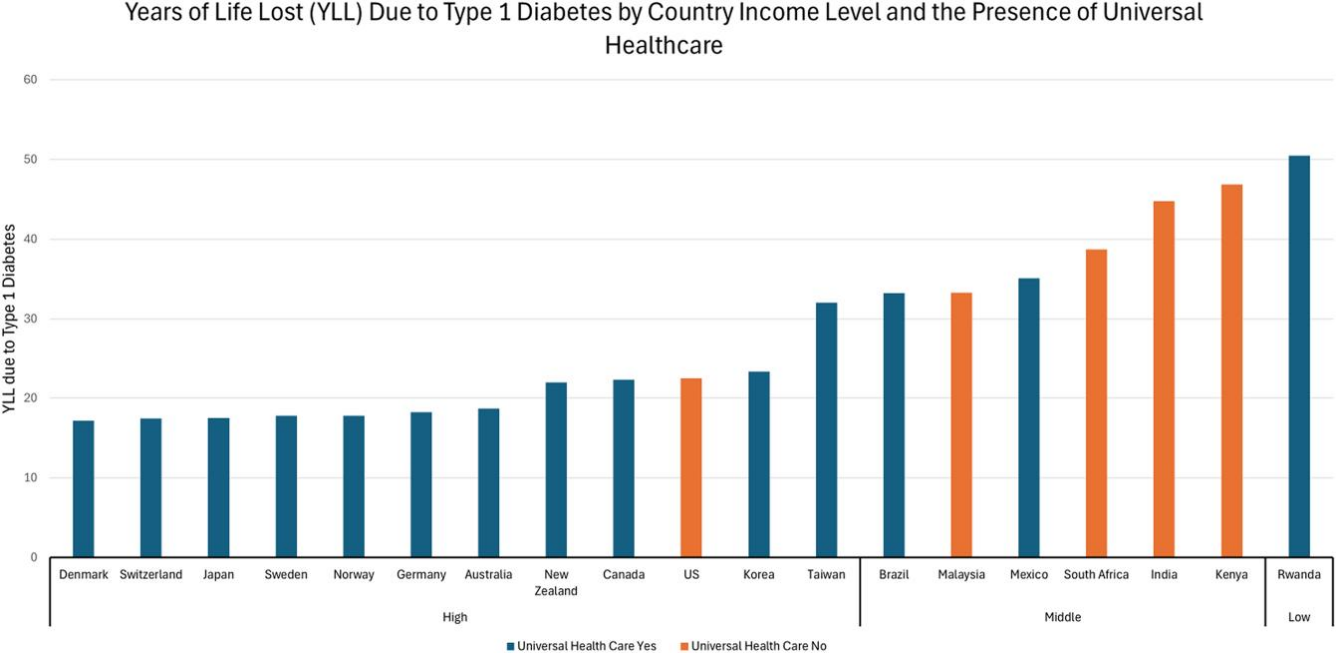
Years of life lost because of type 1 diabetes [1] by income and universal health care coverage of selected countries. Data on years of life lost came from the Type 1 Diabetes Index [1].

The prevalence of diabetes (all types) and T1D specifically as well as the usage of more advanced diabetes technologies in persons with T1D are presented by geographic region for some selected countries around the world in Table 1. The proportion of youth and adults with T1D using advanced diabetes technology varies by region, with a significant number of younger patients in the United States, Australia, and Japan using CGM and insulin pumps, reflecting better access and health care infrastructure, but negligible market penetrance in many Asian, Latin American, and African countries. In many countries, coverage for these devices is more comprehensive for children than adults. Table 2 shows barriers to technology use by country, with many of the barriers similar across countries, and economic barriers being common to all. Data available on YLL due to T1D by high-, medium-, and low-income country status and the existence of universal health care are presented in Fig. 2. Taken as whole, these data underscore the importance of addressing regional disparities in diabetes technology access. Policy initiatives and healthcare planning should focus on improving technology availability and accessibility, especially in lower income countries, to enhance diabetes management and patient outcomes globally.

**Table 1.** Prevalence of type 1 diabetes and usage of diabetes technology by selected countries and regions*^a^*

	Overall prevalence of diabetes (percentage of overall population with diabetes)	Percentage of overall population with diabetes that have type 1 diabetes	Percentage of patients with type 1 diabetes that use medical technology (pump/CSII, CGM, or closed-loop hybrid system/automated insulin delivery system)	Percentage of adolescent and young adult patients with type 1 diabetes that use medical technology (pump/CSII, CGM, or closed-loop hybrid system/automated insulin delivery system)
**North America**				
Canada	5.88 million 15% of population [10]	5%-10% [11]	35% pump 24% CGM [12]	No data
United States	38.4 million 11.6% of population [13]	5%-10% [13]	66% CGM [14] 41% pump [14]	40% CGM [14]
**Latin America**				
Mexico	14.1 million 14.1% of population	6% [15]	21% CSII [15] 9% CGM	No data
**Asia**				
Korea	4.4 million 13.9% of population	1.1%	10.7% CGM [16, 17]	14.0% pump 39.3% CGM [18]
Japan	11.96 million 14.6% of population [19]	2.4% [20, 21]	39% pump 11% SAP 48.4% CGM [22]	21%-28% SAP [22]
Taiwan	2.186 million 11.3% of adults [23]	0.24%	No data	No data
Malaysia	3.6 million 19% of population [24]	0.62% [25]	<10% [26]	No data
India	101 million 11.4% of population [25]	5%-10%	<10% pump No data on CGM	10%-15%
**Africa**				
Rwanda	400 000 [1] 4.5% of population	0.9% [1]	Technology not available	Technology not available
Kenya	460 000 [27]-821 500 [28] 4.2% [27] to 5.1% [28] of population	≤10% [27]	3% CGM [29]	No data
South Africa	4.6 million [30] 10.5% [30] to 11.3% of population [28]	7% [1, 28]	1% pump 16% CGM [29];	No data
**Europe/Australia/New Zealand**				
Australia	1.3 million 5.3% population [31]	9.6% [31]	33.3% pump 80.3% CGM	pump 45% CGM 70% [32]
New Zealand	268 700 6.2% of population	5-10% [33]	No data	For children <15 years: 40% pump 67% CGM 2.3% AID
Switzerland	400 000-500 000 of 8.74 million (∼5% of the population) [28, 34]	0.8%-1% [1]	No data	No national data. One center experience: aged <20 years: 50% pump (SAP or AID) 95% CGM;
Other Europe	61 million 7% of population	5%	Germany: 57% pump 49% CGM [35] Denmark: 39% pump 60% CGM [36] Norway: 74% pump 52% CGM [37] Sweden: 70% pump (children) [38] and 30% pump (adults) [39] 80% CGM [38]	Technology is generally fully covered for children

Abbreviations: AID, automated insulin delivery system; CSII, continuous subcutaneous insulin infusion; SAP, sensor augmented pump.

^*a*^Where available, data came from published literature. All data not cited are from the survey, which included data gathered from country-specific Ministry of Health, country-specific diabetes association, national government database, unpublished data from Australia's National Diabetes Services Scheme (NDSS) data, and diabetes stakeholders and stakeholder groups.

**Table 2.** Barriers to diabetes management technology use among persons with type 1 diabetes*^a^*

Country	Barriers
**North America**	
United States	*Economic barriers* Lack of health insurance/out-of-pocket costs [40, 41] Public (government-funded for the poor or elderly) health insurance may not cover CGM, but if it does, it often requires the patient to meet strict requirements, including daily blood glucose checks [41] *Structural/accessibility barriers* Lack of access to an endocrinologist as many primary care doctors lack the knowledge about diabetes devices and how to manage them [42] *Provider/psychological bias* Provider bias/provider perception of the inability of the patient to manage the device [42] Type of employment may be a barrier, for example work involving heavy physical labor producing perspiration may be an interference [42] Individuals not wanting to have a diabetes device attached to their bodies [41] Concerns about disruptions by alarms or having a device visible on the body [41] Worrying about what others think about the device if worn on the body [41]
**Asia**	
Korea	*Economic barriers* Price burden determines whether insurance is applied It is also expensive, even though it is partially covered by national insurance *Structural/accessibility barriers* There is a problem with the raw data not being received by the provider, except for the Hankook iSense machine *Provider/psychological bias* For CGM, side effects such as skin rashes, dents, and discomfort when detached and reattached every 2 weeks Doctors and nurses have to spend a lot of time educating patients on CSII and CGM use, which is challenging in Korea as this is not financially reimbursed by insurance providers and patients do not want to pay for it About 50% of teenagers hate using technology because they must always wear devices
Japan	*Economic barriers* New devices are still expensive, even if covered by universal health insurance. For type 1 diabetes patients aged 20 years or older, there is no public subsidy beyond the universal health insurance [43, 44] *Structural/accessibility barriers* Limited number of medical institutions and physicians that can deal with the technology Limitation in the accuracy of CGMs compared to SMBG [43, 44] Difficulties and inconvenience of attaching and managing the devices [43, 44] Infusion trouble, skin trouble [43, 44] *Provider/psychological bias* Lack of time for doctors to fully instruct patients on how to safely operate the device and control blood glucose levels [43, 44] Inconvenience of carrying around the device and it not fitting comfortably in daily life [43, 44]. Psychological burden of seeing and not being able to manage widely vacillating glucose levels [43, 44] Perceived or actual prejudice and being discriminated against because of wearing the devices [43, 44]
Taiwan	*Economic barriers* High retail price is the major concern for their usage by the patients for daily monitoring of blood glucose [45]
Malaysia	*Economic barriers* Individuals in Malaysia interested in using diabetes technology devices generally need to bear the costs personally *Provider/psychological bias* Participants’ perceptions of the purpose of SMBG, the emotions associated with SMBG, and the complexity, pain, and cost related to SMBG as well as personal and family motivation are the key concerns or barrier toward using SMBG in Malaysia [46]. Regarding other devices like AID systems and insulin pumps, there is currently a lack of published data on the barriers to their use. Nonetheless, aside from cost, it is anticipated that similar obstacles may apply to AID systems and insulin pumps as well.
India	*Economic barriers* High cost: Diabetes technology devices and treatments can be expensive, making them inaccessible to a large portion of the population in India. The high cost of these technologies can act as a significant barrier for individuals who may benefit from them but cannot afford them *Structural/accessibility barriers* Lack of awareness: Many individuals in India may not be aware of the latest diabetes management technologies available to them. This lack of awareness can prevent them from seeking out these tools and incorporating them into their diabetes management routine Limited access to health care facilities: In many parts of India, access to health care facilities, especially those equipped with the latest diabetes technologies, can be limited. This lack of access can prevent individuals from receiving proper guidance on how to use these technologies effectively *Provider/psychological bias* Cultural beliefs and stigma: Cultural beliefs and stigma surrounding diabetes can also impact the adoption of diabetes technology in India. Some individuals may be hesitant to use these technologies due to cultural beliefs or fear of being stigmatized for their condition Inadequate training for health care professionals: Health care professionals in India may not always receive adequate training on how to use and implement diabetes technologies effectively. This lack of training can result in healthcare professionals being unable to guide patients on the proper use of these technologies Overall, these barriers can collectively hinder the widespread adoption and effective utilization of diabetes technology in India, impacting the overall management and treatment of diabetes in the country.
**Africa**	
Rwanda	*Economic barriers* It is unaffordable and therefore there is negligible market penetrance in the country
**Australia and New Zealand**	
Australia	*Economic barriers* Financial, Socioeconomic status [32] *Structural/accessibility barriers* Potential factors may include distance to health care services, rural internet challenges *Provider/psychological bias* Technology understanding and acceptability, including language/cultural factors [32] Clinician bias
New Zealand	*Economic barriers* Socioeconomic status [47] *Structural/accessibility barriers* Geographic location (rurality) [47] *Provider/psychological bias* Racial provider bias [47]
**Europe**	
Switzerland	*Economic* Financial (out-of-pocket costs) *Structural/accessibility barriers* Insurance: there are some minor restrictions by insurance provider for coverage *Provider/psychological bias* Language/cultural factors Clinician perception of the inability of the patient to manage the device PwD not wanting to wear a device on the body/stigma. Low education status of PwD may influence adoption of technology

Abbreviations: AID, automated insulin delivery system; CSII, continuous subcutaneous insulin infusion; PwD, person with diabetes; SAP, sensor augmented pump; SMBG, self-monitoring of blood glucose by needle finger prick.

^*a*^Where available, data came from published literature. All data not cited are from the survey.

### North America

The United States and Canada are 2 high-income countries with among the highest rates of T1D incidence in the world. Of high-income countries, they also rank next to the highest for YLL because of T1D. Despite having very different health care models, they have a similar 22 to 23 YLL from diabetes [1]. This lack of an expected difference in YLL given the very different health care models may be due to differences in utilization of advanced diabetes technology. Canada has universal health coverage, whereas the United States does not; however, use of diabetes technologies such as CGMS, CSII, and AID is higher in the United States [12, 14].

#### Canada

There are 5.88 million people in Canada who have diabetes [10]. It is estimated that 5% to 10% of diagnosed people with diabetes in Canada have T1D with an estimated population prevalence of 250/100 000 [11], and one of the highest incidence rates globally. However, despite having universal health care, only about one-third of youth with T1D use advanced diabetes technology; YLL from diabetes is at the upper end among the high-income countries surveyed for this report.

The use of diabetes technology in Canada is supported in part by provincial health plans, but sometimes only for children or young adults. Therefore, coverage of the expense for adults can require private health insurance or coverage through other entities, such as Diabetes Canada [48]. The Diabetes Canada guidelines on blood glucose monitoring in adults and children with diabetes recommend the use of real-time or intermittently sampled CGM for people with T1D using multiple daily injections or insulin pumps [49]. However, in a 2023 study of glycemic management in 306 children and youth with T1D, CGM was reported in only 24% and CSII use in 34.6% of participants [12]. The use of CGM was lowest among the 2 highest quintiles of socioeconomic deprivation (16.8%) compared to the least deprived 2 quintiles (31.3%, *P* = .03). Similarly, CSII use was lowest in the most deprived 2 quintiles (23.8%) compared to the least deprived 2 quintiles (42.5%).

#### United States

It is estimated that in the United States, 1.7 million adults and 304 000 children and adolescents have T1D [50]. Globally, the United States has the second highest incidence of T1D among children and adolescents younger than age 20 years [28]. Although the United States is a high-income country that does not have universal health insurance, the percentage of persons with T1D using diabetes technology is in the mid-range compared to other high-income countries. However, its 22.5 YLL resulting from T1D is in the upper range among other high-income countries (Fig. 2). Technology access and usage in the United States varies by insurance coverage and type, socioeconomic status (SES), and independently of SES by race/ethnicity [51-54].

The Type 1 Diabetes Exchange Quality Improvement Collaborative (T1DEX-QI) examined use of CGM among more than 11 000 patients with T1D seen at a mixture of pediatric and adult diabetes clinics across the United States [14]. Among adolescents and young adults aged 13 to 26 years, 40% used CGM. This was lower than the prevalence of CGM use in pediatric patients aged 2 to 12 years (52%) and in adults 27 to 55 years (66%) and aged older than 55 years (56.5%) [14]. Overall, in this cohort, CSII were used by 41%, and use of both CGM and CSII were reported for 30% [14].

Diabetes technology use varies by race/ethnicity and by income and socioeconomic status in the United States. In the T1DEX-QI cohort, CGM use among all patients was highest in non-Hispanic White (NHW) patients (49.5%), lowest in non-Hispanic Black (NHB) patients (17.7%), and intermediate in Hispanic patients (38.4%) [14]. In data reported from a single specialty diabetes clinic, when compared by age group, use of any diabetes technology among youth was 93.5% in NHW vs 55.1% in NHB and 77.5% in Hispanic patients; among adults, these racial and ethnic disparities were less marked, with 85.6% of NHW patients reporting any technology use compared to 63.4% of NHB and 66.9% of Hispanic patients [51].

Health insurance often covers diabetes technology in the United States, though there can still be high out-of-pocket costs and significant barriers to use even when insurance coverage is provided. Consistently, data show lower rates of diabetes technology use among those with public insurance compared to private insurance. In the T1DEX-QI, among all patients CGM use was higher in those patients who had private insurance (57.2%) compared to patients who were on government insurance (33%) [14]. In Colorado youth aged 12 to 17 years with T1D, Medicaid insurance was associated with a 65% reduced odds of using any diabetes technology, a 59% reduction in odds of using an CSII, and a 60% reduced odds of using CGM compared to private insurance, despite the fact that Medicaid in Colorado covers these devices [51]. A Medicaid supplement through the California Children's Services provides CGM coverage, but children wanting to use CGM under this program have to check blood glucose ≥4 times a day to obtain coverage, and those wishing to use CSII also have to demonstrate consistent CGM use [40]. Setting these types of requirements for diabetes device approval under public insurance not only creates disparities in use of these devices, given that private insurance does not carry these requirements, but also prevents the use of diabetes technology by those who could most benefit. In a case series, improved management of diabetes, more sustained use of CGM systems and improvement in glycemic control were reported among youth on public insurance when these barriers to use were removed [40].

In addition to lack of insurance coverage, lack of access to an endocrinologist can be a major barrier to obtaining and using diabetes technologies [42] because many primary care doctors lack the knowledge about diabetes devices to manage them; provider bias can also present a barrier [42]. Individual-level barriers are also often reported, including having difficulty wearing devices because of work conditions [42]. In adults with T1D who took part in the T1D Exchange, almost half reported that the hassle of wearing devices was a major barrier, and more than one third did not like having diabetes devices on their body [41]. There were also significant concerns about the device not working or relying on the technology, taking more time to manage their diabetes, and worrying about what others think about the device [41].

### Latin America

Latin America includes 21 countries and has a total population of almost 500 million. The prevalence of T1D ranges from 0.4 to 8.3 cases per 100 000 in children younger than age 15 years. In many places, only a minority of individuals currently receive treatment with advanced insulin infusion or glucose monitoring therapy for T1D. Only 2% of persons with T1D in Latin America use AIDs [55].

Although some of the differences in diabetes technology utilization in Latin America can be explained by social inequalities, economic and ethnic differences among Latin countries, and long-term structural differences within the health care system models [56, 57], for most countries in Latin America, low SES restricts access to technologies for diabetes treatment. Persons living with T1D in Latin America face several challenges, including lack of universal health care coverage in many countries, low national health expenditure for T1D, and limited access to appropriate glucose management regimes. Some country-specific examples reported on next include Mexico and Brazil.

Brazil, a middle-income country, has the third highest incidence globally for T1D among children and adolescents younger than age 20 years. All ages combined, more than half a million people are living with T1D in Brazil. However, use of more advanced diabetes technologies such as CGM, CSII, or AID is still very limited despite the majority of the population receiving free health care through its government-funded health care system. CGM accessibility via the Brazilian Public Healthcare System is limited [58, 59]. It is estimated that among persons with T1D in Brazil, an average of 33 years of healthy life years are lost [1].

Mexico, a middle-income country bordering the United Sates at its southern boundary, has an estimated 90 000 people living with T1D [1, 15]. Though a middle-income country bordering the very high-income country the United States, there is very little information regarding treatment, including diabetes technology. However, in a study by RENACED-DT1, a national type 1 diabetes patient registry representing a small sample of persons with T1D, of 964 patients from 29 clinics in its registry, 21% of the patients were using CSII and 9% were using CGM [15]. YLL from diabetes is estimated to be 35 years.

### Asia

The lowest rates in the world for T1D incidence are found in Asia, in particular China, Japan, and Korea; however, because of the population size of certain Asian countries, in particular China and India, Asia also contains the countries that account for the largest numbers of persons with T1D. Because Asia accounts for the largest numbers of T1D in the world, the lack of diabetes technology access in this region may potentially have the greatest negative impact globally [60].

#### Japan

Japan has one of the lowest incidence rates of T1D in the world [61-63]. According to the Ministry of Health, Labour and Welfare's 2020 patient survey, the total number of patients of all ages receiving continuous medical care for T1D was 139 000, with a prevalence rate of 0.11% of the overall population and 2.4% of the overall population with diabetes [20]. Japan is a high-income country with universal health care that covers CGM, CSII, and SAP. Additionally, there is public subsidy for these devices available to T1D patients younger than age 20 years. There is a report on the use of diabetes technology in multicenter cohort study of pediatric patients with T1D. Overall, approximately 50%, 40%, and 10% of children and adolescents with T1D in Japan use CGM, CSII, and SAP, respectively. By age group, the usage is lower among adolescents [22].

Japan's universal health insurance covers diabetes technology for T1D patients also aged 20 years or older; however, there is no additional public subsidy (ie, government-funded coverage of health care) for them. Thus, especially for adults, the largest barrier to diabetes technology use is cost. Other barriers to technology use include difficulties and troublesome annoyance of attaching and managing the devices, the psychological burden of seeing and not being able to manage widely vacillating glucose levels and a limitation in the accuracy of CGMs compared to self-blood glucose monitoring (SMBG). For adolescents in particular, additional barriers include not wanting to be seen as different from their peers by attaching the device, the inconvenience of carrying around the device, and it not fitting into their daily life [43, 44].

#### Korea

Korea also has one of the lowest rates in the world for T1D; however, data show that incidence rates have been increasing. Korea is also a high-income country with universal health care; however, until 2024, CGM was not covered, except for in children and adolescents younger than age 19 years. However, 93% of the T1D population in Korea is aged 19 years or older [16]. Although use of CGMs and pumps is low, both are partially covered by insurance and the use of CGMs is increasing. Choe et al reported the use of CGMs increased from 1.4% in 2010 to 39.3% in 2019 and the use of CSII increased from 2.1% to 14.0% from 2010 to 2019 in a multi-(academic)-center study of children and adolescents in Korea [18]. However, the Korean National Health Insurance Service (NHIS) has 18.7% of children younger than age 20 years registered as using CGMs [18]. As of December 2022, 10.7% of the entire T1D population registered in the Korean NHIS were using CGMs [16, 17].

The majority of persons with T1D in Korea practice multiple daily injections with an insulin pen and SMBG with a finger prick glucometer. Only 0.4% of persons with T1D in Korea use an AID [16], largely because of the out-of-pocket cost burden to the patient or family with T1D. For children younger than age 19 years, insurance will pay for 70% of the cost of AID, but the cost of this technology is high. For adults with T1D, Korean NHIS will pay for 70% of the cost of sensors for CGMs and approximately 50% of pumps [17], still leaving considerable out-of-pocket costs. Further, patients have to pay for the technology up front and then receive reimbursement for the percentage that the insurance will cover. In addition to the financial costs, barriers to technology use include side effects such as skin rashes, skin dents, and discomfort when the CGM or AID is removed and reattached every 2 weeks. Both in children and adults in Korea, CGM use is associated with improved glycemic control [18, 64].

#### Taiwan

Like Korea and Japan, the incidence of T1D in Taiwan is one of the lowest in the world, with only 0.24% of the population with diabetes having T1D [65]. This is reflected in the scarcity of studies on T1D in Taiwan and no published data on diabetes technology use. CGM was introduced in Taiwan on March 5, 2021, so the market share for CGM is small. Additionally, although Taiwan does have universal health insurance, requirements for coverage of CGMs are strict, limiting the growth of the usage of CGMs in this country, and CSII is not covered at all. The high retail price of diabetes technology and their subsequent out-of-pocket cost is the major barrier to diabetes technology usage in Taiwan, a high-income country where YLL from diabetes is similar to that of medium income countries rather than that of other high-income countries.

#### Malaysia

The estimated prevalence of T1D in Malaysia when extrapolated from National Diabetes Registry data is 0.62% of all persons with diabetes [25], one of the lowest prevalence rates in the world. More than 50% of T1D patients aged younger than 20 years old have poor glycemic control with an HbA1c of more than 10.0% [26]. For all persons with T1D, both adults and children, less than 10% use diabetes technology. YLL from diabetes is 33.3 years.

Few data are available on diabetes technology use in Malaysia, but it is reported that CGM and CSII have been available in Malaysia since 2005. CGM has been incorporated in many local diabetes-related clinical research centers and its use in routine clinical practice has also increased in recent years [66]. Nevertheless, <10% of people with T1D in Malaysia use CGM [26]. CSII therapy is initiated primarily among adolescent patients with T1D with poor control on multiple-dose insulin injections. As of 2015, there were approximately 170 local patients using CSII therapy, with an estimated 70% having T1D. Use of CSII has been increasing in recent years, with up to 30 to 40 new patients initiated per year. In Malaysia, the use of CSII or CGM is not funded by the government nor is it covered by insurance; therefore, patient selection is done in accordance with the specific indications as outlined in the established local clinical practice guidelines [67].

Research investigating barriers to diabetes technology uptake in Malaysia, particularly among individuals with T1D, is limited. An existing study, primarily focused on type 2 diabetes, indicates that cost of glucose strips, frustration related to high blood glucose readings, misperception, stigma, fear of needles, inconvenience, lack of motivation, and lack of self-efficacy are the common barriers for SMBG [46]. Additionally, for CGM, the highlighted key barriers include high cost, technology complexities, and inadequate training on the use of the device. The lack of device training may be attributed to the lack of trained diabetes educators and short consultation time with clinicians in most public health care settings.

#### India

According to estimates from the International Diabetes Federation, India has the highest number of cases of T1D in the world, adults and children inclusive. It is estimated that 128 500 children and adolescents in India have T1D [68]. However, only 10% to 15% of persons with T1D in India use diabetes technology to manage glycemia. YLL from T1D is 44.8 years. Thus, not only does India account for the greatest number of people with T1D, it also is one of the countries with the highest documented YLL due to T1D.

A recent study examined whether use of CGM improved glycemic control in children with T1D in resource-poor areas in India and found that baseline HbA1c was 11.23% in the intervention group and 11.62 in the control group, demonstrating very poor glycemic control. The use of CGM reduced HbA1c significantly in the intervention group, to 10.14% (*P* = .01), with no significant change in the control group (follow-up HbA1c 11.32%) [69]. Similarly, a study examined CSII use in 50 children with T1D who were from rural, below-poverty families. The use of CSII therapy in these children significantly improved glycemic control, from a mean HbA1c of 10.42% before pump initiation to 7.5% after 1 year of therapy [70].

The Indian Council of Medical Research issued guidelines for the management of T1D in 2022 [71] and recommended glucose monitoring by SMBG because glucometers, but not CGM, are widely available in India. CGM use is recommended for people with hypoglycemia unawareness and for motivated patients (those who are willing to wear the device at least 70% of the time). India does not have universal health care and the out-of-pocket cost of technology such as CGM may be prohibitive for most patients with T1D, particularly if insurance does not cover it. Additionally, use of CSII may be hampered, particularly among low-income patients, by myths surrounding its use, including that rural and poor families will be unable to effectively use CSII, that pumps do not work well in the hot weather, and that they are mostly for wealthy and literate people [70].

### Africa

Some of the highest rates for incidence and prevalence of T1D are found among African countries, both northern and sub-Saharan Africa. According to the International Diabetes Federation [28], Eritrea ranks first and Libya ranks ninth in the world for the incidence rate of adult-onset T1D, whereas Algeria ranks fourth and fifth for incidence by count (number of new cases) and prevalence among children younger than age 10 years; Morrocco ranks sixth for incidence and prevalence, and Nigeria ranks eighth for incidence among children younger than age 20 years. However, for most countries in Africa, market penetrance of diabetes technology is negligible and the continent reports among the highest years of life lost because of T1D.

Throughout the continent of Africa, even in middle-income countries, use of advanced diabetes technology is scant to nonexistent due to the price burden for patients and the health system. For example, data from FIND market research showed that in South Africa and Kenya, 2 medium-income countries in Africa, the prevalence of CGM usage among persons with T1D is 16% and 3%, respectively. Most countries in Africa are low-income countries. In most countries in Africa, point-of-care testing devices, access to insulin (and its storage) and more basic diabetes technology such as a finger stick glucometer and testing strips, are the more pressing challenges [72, 73]. Cost of insulin, a lifesaving essential medicine, could account for up to half of a household's annual gross income. While globally, only 1 of 2 people in need of insulin have adequate access to it either because of availability or affordability, in Africa this is much lower, with only 1 in 7 persons in need of insulin having adequate access to it [74]. In many countries, half of newly diagnosed persons with T1D die within the first year of diagnosis. This broader issue of access to insulin has been highlighted by Virmani et al [75]. Universal health care does seem to mitigate some of these challenges, including with diabetes technology. For example, although CGMs are not available in Rwanda because of their cost, Rwanda a low-income country, does have universal health care, and approximately 90% of adolescents and young adults with T1D use a home glucometer.

### Australia and New Zealand

In Australia, data from a center in Western Australia, where 10% of the national population resides, estimated that 93% of children and adolescents diagnosed with T1D are using CGM and 57% use CSII. Although Australia does have universal health care that covers CGM for all persons with T1D, this does not include CSII. Barriers to technology use include the costs associated with diabetes technology use, specifically CSII use given access in Australia is through top-tier private health insurance, self-funding, or limited philanthropic programs, SES, and the distance to health care services. Despite universal access to CGM, a 2023 report by Lomax et al found that disparities in diabetes technology use in Australia still existed [76]. Those in the quintiles associated with the highest socioeconomic disadvantage were the least likely to use diabetes technology for both CGM and pump. CGM use was higher and more even across the other quintiles, whereas pump use increased in each quintile associated with less socioeconomic disadvantage. In addition to SES and technology associated costs, barriers to technology use in Australia were hypothesized to include clinician based bias, access to relevant supporting technology (for example, mobile devices), technology acceptability, and understanding including impacts of cultural and language factors [32, 76].

Approximately 5% of the New Zealand population with diabetes have T1D (personal communication, Martin de Bock, MD, PhD, University of Otago). Like Australia, New Zealand has universal health care; however, while it does cover CSII contingent on certain health behavioral characteristics, it does not cover CGM. Starting October 1, 2024, the restrictions on who can be covered for CSII use will be removed and CGMs and AID systems will be covered. Thus, starting October 1, 2024, these more advanced diabetes technologies will become free to all persons with T1D in New Zealand. Hopefully, this will help reduce the disparity in technology use shown to exist by SES in New Zealand [47] and mitigate glycemic disparities by ethnicity [47]. In explaining these disparities by race/ethnicity in New Zealand, Burnside et al suggested that provider bias in ethnicity-based treatment as well as specific ethnicity and cultural factors may contribute [47].

### Europe

There are an estimated more than 2.9 million people in Europe with T1D [60], among them an estimated 295 000 children and adolescents. For most European countries, survival with T1D surpasses that of most countries of the world, including other high-income countries such as the United States [1]. Compared to other countries and regions of the world, including the United States, usage of advanced diabetes technology such as CSII, CGM, and AID is generally higher in European countries.

#### Germany, Austria, Luxembourg, and Switzerland

The Diabetes Prospective Follow-up (Diabetes-Patienten-Verlaufsdokumentation [DPV]) registry is an initiative for quality improvement and captures data from diabetes centers in Austria, Germany, Luxembourg, and Switzerland. It covers more than 80% of all pediatric patients with diabetes in Germany, Austria, and Luxembourg [77], but not Switzerland.

In the 2016 through 2018 period, insulin pump use was 56.6% and CGM use 48.7% among German youth with T1D [35]. CGM use decreased across quintiles of SES, with 48.5% of those in the lowest SES quintile using CGM in 2016 through 2018, compared to 57.1% in the highest SES quintile (*P* < .0001); in contrast, insulin pump use did not differ across SES quintiles (*P* = .4109) [35]. Similar pump and CGM use rates were observed in a single center in Germany with insulin pump use in 63.1% overall, with lower use in adolescents 12 to 17.9 years of age and young adults 18 to 20.9 years than in younger children [78]. CGM use was reported by 31.6% overall but was highest in the 12- to 17.9-year age group (35.3%), and lower in young adults aged 18 to 20.9 years (28.6%) [78]. A marked increase was observed in CGM use since 2016 through 2017, when intermittently scanned CGM (isCGM) became readily available and CGM was funded for people with T1D in Germany. Subsequently, CGM uptake (intermittently scanned CGM [isCGM] and real time CGM [rtCGM]) increased exponentially [79]. In 2020, CGM use increased to 76% in a sample of more than 32 000 children and young adults in the DPV registry [79]. It varied by age, with 76% of children younger than age 6 years using CGM, and 58% of young adults aged 18 to 25 using CGM [79]. In a study of more than 13 000 adults in the DPV registry, in 2019 through 2021, use of insulin pumps was 56.1% and CGMs 75.3% in 18- to 25-year-olds, and decreased with older age [80]. Adults residing in Germany who had a migration background were less likely to use an insulin pump (27.6%) or a CGM (61.4%) than those without a migration background (insulin pump [38.8%], CGM [71.1%]; *P* < .0001 for both) [80]. Use of diabetes technology also decreased along quintile of area deprivation, although this relationship was not linear [80].

Single-center experiences in Switzerland show a similar increase in diabetes technology use in pediatric patients following the availability and funding of isCGM systems since 2017. In 2017 through 2018, in a single-center cross-sectional study of children and adolescents with T1D followed in the outpatient clinic in Bern, 26.4% used an insulin pump and 39.3% used a CGM [81]. Similarly, in a different Swiss center pump use increased from 33% in 2018 to approximately 50% in 2023 and CGM use increased from just under 50% to around 92% to 95% over the same years (personal communication, Marie-Anne Burckhardt).

#### Denmark, Sweden, and Norway

In a study of more than 2000 adults with T1D in Denmark, 38.6% used an insulin pump and 60% used a CGM in 2020 [36]. Sweden has provided coverage for CGM since 2014, although it was initially limited to only those with inadequate glycemic control [82]. The reimbursement has now widened, and 79.6% of participants with T1D in the Swedish National Diabetes Register used CGM in 2019. As of 2020, 70% of children in Sweden used insulin pump therapy [38]. In contrast, only 30.1% of adults with T1D in Sweden use an insulin pump, with highest use among 18 to 21 year olds (45.2%), intermediate use in 22 to 30 year olds (31.3%) and lowest use in adults older than age 30 years (22.3%) [39]. In Norway, all diabetes technologies are available at no cost for children who have T1D, while adults have to pay an annual deductible equal to approximately €265 [83]. As a result, Norway already had very high use of insulin pumps (74%) and CGM (52%) among pediatric patients in 2017 [37].

## Discussion of Common Themes

Universally, the most common barrier to technology adoption and utilization, irrespective of the country's economic status, is the financial burden. For many middle- and low-income countries, this financial strain means zero market penetrance of the technology into the country. Due to their prohibitive cost, both technology companies and national governments may lack incentive to pursue market entry into these regions. In countries where the market penetrance exists, inadequate insurance coverage or high up front out-of-pocket costs for the individual patient or families with diabetes also hinder the adoption and sustained usage of these advanced technologies.

Another common structural economic theme across countries is that, even when full or nearly full coverage for technology is available, it is often restricted to the pediatric population. Consequently, a significant proportion of patients in late adolescence or young adulthood with T1D who are either still in school or just beginning to earn income, are left without access to affordable diabetes technologies.

Other structural barriers to diabetes technology such as stringent eligibility criteria imposed by many insurance providers, whether private or government-based, further impede access to diabetes technology. This has been shown to harm those most in need because they have to demonstrate optimal glycemic control prior to the approval for the technology.

Furthermore, lack of awareness or insufficient knowledge of the technologies by both patients and providers is another common barrier across many countries. Notably, even in high-income countries, many health care providers lack knowledge on utilization of these diabetes technology devices, and some may fear legal liability due to inadequate patient education.

Among high-income countries with a diverse racial and ethnic population, such as the United States and New Zealand, disparities by race and ethnicity have been observed. This often stems at the level of the health care provider and persists even in the setting of equalized income or universal health care coverage. For example, a study in the United States showed that African American pediatric patients with T1D whose family household incomes were at least $100 K per year were prescribed advanced diabetes technology at a much lower rate than White children from similarly incomed families but at the same rate of White pediatric patients whose family household annual income was $50 000 or less per year. Disparities by race/ethnicity unaccounted for by SES or insurance have also been reported in Australia and New Zealand [32, 47, 76].

Finally, disparity in the uptake of diabetes technology is also compounded by individual-level barriers [84]. One of the common individual-level barriers that has been widely cited is psychological burden. Results of a recent survey among individuals with T1D exploring barriers in adoption of CSII and CGM showed that nearly half of respondents reported device inconvenience, whereas one-third expressed discomfort with device attachment onto their body [85]. Some individuals reported negative impact on their body image due to constant presence of a device on their body [86]. Self-reported diabetes stigma is also prevalent, with many individuals perceiving wearing diabetes technology as a personal failure or burden, contributing to psychological distress. Additional glycemic information from devices such as CGM can be overwhelming or result in “information overload,” prompting users to discontinue its use [87]. Some individuals also experience frustration with frequent alarms and technical issues. Moreover, fears, such as dependency on technical devices, and difficulty trusting devices, may further impede the uptake of diabetes technologies [88].

## Limitations and Future Directions

We acknowledge that countries used in this mini-review comprised a convenience sample. This was not a meta-analysis, nor was it a systemic review. We conducted a convenience sample to help gather the data to obtain a global perspective on disparities in technology use in T1D, as this information is not generally available for very many countries globally. As such, in addition to increasing market penetrance of diabetes technology into low- and middle-income countries, and its affordability to individuals in all countries, our research for this mini-review suggests some potential future directions:

- Several countries, especially in Latin America, Africa, and parts of Asia, have incomplete data on T1D prevalence and technology usage. This highlights the need for more comprehensive and updated studies to better understand the global landscape of T1D management.
- In many countries, most epidemiological studies on T1D to date have focused on children. For example, there are no data on the incidence of T1D in adults in Japan. It would be desirable to have a system in which new-onset patients (both children and adults) are registered so that they can be accurately captured.
- A survey of national medical databases in countries where they exist, for example Japan's National DataBase, which is a database of information on receipts and specific health checkups, would be effective for identifying T1D patients of all ages and their treatment status. With this, it would also be necessary to examine and validate the logic, that is, the algorithms, for accurately extracting T1D from the receipt data.
- Continued research and surveillance using large data sets and registries, but with an equity lens, to ensure that those who are most disadvantaged are not left behind due to systemic barriers to care even where there is free access to modern diabetes technology.
- Studies often lack diversity in participant demographics, limiting the generalizability of findings. In view of this, particularly large data sets should be examined for the impact of socioeconomic factors, health literacy, and cultural barriers on technology adoption and outcomes.
- Few studies explore strategies to reduce cost barriers or improve insurance coverage. This is an important area that needs more research.
- The role of health care provider biases in prescribing technology and the impact of digital health literacy on equitable access remains largely underexplored. This also is an area that needs more research.

## Conclusion

Type 1 diabetes is a severe, life-threatening disease resulting in an average of 32 YLL globally, seen most drastically in middle- and low-income countries [1]. Although the incidence of T1D is not affected by SES, the development of complications and limited access to modern therapy is overrepresented in vulnerable populations [89]. Diabetes technology, particularly CGM, has been shown to greatly improve glycemic management, and is therefore crucial to the long-term impact and potentially survival of patients with T1D. To ensure equitable access, major diabetes technology manufacturers and distributors should work toward lowering the cost of diabetes technologies in middle- and low-income countries. National governments and private health insurance companies worldwide should mitigate the barriers to access including high out-of-pocket cost, complex reimbursement schemes, stringent eligibility or indication criteria for devices, and insufficient funding for education on technology usage. Additionally, efforts to address racial and ethnic disparities in technology usage, especially in high-income countries, should be intensified targeting physician bias awareness and patient education in vulnerable groups. Finally, psychological and cultural barriers to technology use at the individual level should be explored by the health care teams and appropriate education and support provided. This review was written as a joint effort between diabetes epidemiologists, physicians, laboratory-based clinicians, and diabetes medical technology manufacturers. Our review underscores the need for a joint effort involving national governments, private health insurers, technology manufacturers, and health care providers to address the disparities of diabetes technology utilization worldwide.

## Disclosures

R.B.C., J.S.-B., and A.G.G. have received a grant from the American Diabetes Association to investigate racial disparities in diabetes technology among adolescents with type 1 diabetes. They have no other disclosures to declare. M.d.B. has received research funding from Novo Novonordisk, Medtronic, Ypsomed, Dexcom, and Insulet Honoraria, travel expenses or speaking fees from Novo Nordisk, Sanofi, Pfizer, Medtronic, Boerhinger Ingelheim, Ypsomed, Dexcom, and Insulet, and has served on Advisory Boards for Tandem and Dexcom. M.-A.B. received honoraria for lectures from Medtronic not related to this publication. C.-N.L. is employed by EPS Bio Technology Corporation. A.K.P. is Chief Technical Consultant of Cgenic Meditech Pvt Ltd. W.K. is president of CareforU and owns shares in CareforU. K.H. is Chief Medical Officer and VP of CareforU, a medical technology manufacturing company. The remaining authors do not have any disclosures to declare.

## Data Availability

Original data generated and analyzed during this study are included in this published article or in the data repositories listed in References.

## Abbreviations

AID: automated insulin delivery
CGM: continuous glucose monitor
CSII: continuous subcutaneous insulin infusion
DPV: Diabetes-Patienten-Verlaufsdokumentation
isCGM: intermittently scanned continuous glucose monitor
NHB: non-Hispanic Black
NHIS: National Health Insurance Service
NHW: non-Hispanic White
SAP: sensor augmented pump
SES: socioeconomic status
SMBG: self-blood glucose monitoring
T1D: type 1 diabetes
T1DEX-QI: Type 1 Diabetes Exchange Quality Improvement Collaborative
YLL: years of life lost

## References

[1] Type 1 Diabetes Index . Juvenile Diabetes Research Foundation. Accessed May 5, 2024. https://t1dindex.shinyapps.io/dashboard/

[2] Shah VN, Akturk HK, Joseph H, Schneider N, Snell-Bergeon JK. A randomized controlled trial of transition from insulin pump to multiple daily injections using insulin degludec. Diabetes Obes Metab. 2021;23(8):1936‐1941. 34180122 10.1111/dom.14423

[3] Dubé MC, Lavoie C, Weisnagel SJ. Glucose or intermittent high-intensity exercise in glargine/glulisine users with T1DM. Med Sci Sports Exerc. 2013;45(1):3‐7. 22895370 10.1249/MSS.0b013e31826c6ad3

[4] Fonseca VA, Grunberger G, Anhalt H, et al CONTINUOUS GLUCOSE MONITORING: A CONSENSUS CONFERENCE OF THE AMERICAN ASSOCIATION OF CLINICAL ENDOCRINOLOGISTS AND AMERICAN COLLEGE OF ENDOCRINOLOGY. Endocr Pract. 2016;22(8):1008‐1021. 27214060 10.4158/EP161392.CS

[5] Maahs DM, Chase HP, Westfall E, et al The effects of lowering nighttime and breakfast glucose levels with sensor-augmented pump therapy on hemoglobin A1c levels in type 1 diabetes. Diabetes Technol Ther. 2014;16(5):284‐291. 24450776 10.1089/dia.2013.0227

[6] Yardley JE, Iscoe KE, Sigal RJ, Kenny GP, Perkins BA, Riddell MC. Insulin pump therapy is associated with less post-exercise hyperglycemia than multiple daily injections: an observational study of physically active type 1 diabetes patients. Diabetes Technol Ther. 2013;15(1):84‐88. 23216304 10.1089/dia.2012.0168

[7] Berget C, Akturk HK, Messer LH, et al Real-world performance of hybrid closed loop in youth, young adults, adults and older adults with type 1 diabetes: identifying a clinical target for hybrid closed-loop use. Diabetes Obes Metab. 2021;23(9):2048‐2057. 34010499 10.1111/dom.14441

[8] Akturk HK, Giordano D, Champakanath A, Brackett S, Garg S, Snell-Bergeon J. Long-term real-life glycaemic outcomes with a hybrid closed-loop system compared with sensor-augmented pump therapy in patients with type 1 diabetes. Diabetes Obes Metab. 2020;22(4):583‐589. 31789447 10.1111/dom.13933

[9] Garg SK, Weinzimer SA, Tamborlane WV, et al Glucose outcomes with the in-home use of a hybrid closed-loop insulin delivery system in adolescents and adults with type 1 diabetes. Diabetes Technol Ther. 2017;19(3):155‐163. 28134564 10.1089/dia.2016.0421 PMC5359676

[10] Diabetes in Canada: Backgrounder. Diabetes Canada; 2023. https://www.diabetes.ca/DiabetesCanadaWebsite/media/Advocacy-and-Policy/Backgrounder/2023_Backgrounder_Canada_English.pdf

[11] Nakhla M, Simard M, Dube M, et al Identifying pediatric diabetes cases from health administrative data: a population-based validation study in Quebec, Canada. Clin Epidemiol. 2019;11:833‐843. 31572014 10.2147/CLEP.S217969 PMC6750203

[12] Simba S, Von Oettingen JE, Rahme E, Ladd JM, Nakhla M, Li P. Socioeconomic disparities in glycemic management in children and youth with type 1 diabetes: a retrospective cohort study. Can J Diabetes. 2023;47(8):658‐664.e2. 37481125 10.1016/j.jcjd.2023.07.005

[13] CDC . National Diabetes Statistics Report. Center for Disease Control and Prevention. 2024. Updated May 15, 2024. 2024. https://www.cdc.gov/diabetes/php/data-research/

[14] DeSalvo DJ, Noor N, Xie C, et al Patient demographics and clinical outcomes among type 1 diabetes patients using continuous glucose monitors: data from T1D exchange real-world observational study. J Diabetes Sci Technol. 2023;17(2):322‐328. 34632823 10.1177/19322968211049783 PMC10012384

[15] Faradji-Hazán RN, Valenzuela-Lara M, Díaz-Barriga Menchaca AP, et al Type 1 diabetes care in Mexico: an analysis of the RENACED-DT1 national registry. Rev Invest Clin. 2021;73(4):222‐230. 33944861 10.24875/RIC.20000498

[16] Ahn HY . Telling patients to use a class 4 medical device insulin pump on their own? That's ridiculous. *Health Kyunghyang Magazine*, January 11, 2024.

[17] Kim JH . Current status of continuous glucose monitoring among Korean children and adolescents with type 1 diabetes mellitus. Ann Pediatr Endocrinol Metab. 2020;25(3):145‐151. 32871645 10.6065/apem.2040038.019 PMC7538300

[18] Choe J, Won SH, Choe Y, et al Temporal trends for diabetes management and glycemic control between 2010 and 2019 in Korean children and adolescents with type 1 diabetes. Diabetes Technol Ther. 2022;24(3):201‐211. 34704794 10.1089/dia.2021.0274

[19] The National Health and Nutrition Survey (NHNS) Japan, 2019 Summary. Ministry of Health, Labor and Welfare; 2023. https://www.mhlw.go.jp/stf/seisakunitsuite/bunya/kenkou_iryou/kenkou/eiyou/r1-houkoku_00002.html

[20] Japan's Ministry of Health Law . *Overview of the 2020 Patient Survey*. Ministry of Health, Labour and Welfare; 2024. https://www.mhlw.go.jp/toukei/saikin/hw/kanja/20/index.html

[21] *2020 Patient Survey by Ministry of Health, Labour and Welfare*. Japan's Ministry of Health, Labour and Welfare; 2022.

[22] FY2022 Health and Labour Science Research Grants . (Comprehensive Research Project for Cardiovascular Diseases, Diabetes and Other Lifestyle Related Diseases) “Research to Understand the Actual Condition of Diabetes and to Improve the Environmental Improvement” (Principal Investigator: Toshimasa Yamauchi) Report of General and Shared Research Project (2023). Japan's Ministry of Health, Labour and Welfare; 2023.

[23] Statistics of Health Promotion 2021 . Taiwan. Accessed August 19, 2024. https://www.hpa.gov.tw/EngPages/Detail.aspx?nodeid=3850&pid=17613

[24] International Diabetes Federation . *Malaysia Diabetes Report 2000-2045. IDF Diabetes Atlas*. 10th ed. International Diabetes Federation; 2021. Accessed September 15, 2024. https://www.diabetesatlas.org/data/en/country/120/my.html

[25] Chandran A, Abdullah MN, Abdul F. *National Diabetes Registry Report, 2013-2019*. Division of Disease Control, Ministry of Health, Malaysia; 2020.

[26] Mohd Nor NS, Anuar Zaini A, Jalaludin MY. Self-care management among children and adolescents with diabetes mellitus in Malaysia. J Child Health Care. 2024;28(4):804‐814. Doi: 10.1177/13674935231168911 37029637

[27] Diabetes Kenya . Ugonjwa Wa Sukari Kenya. Accessed October 1, 2024. https://diabeteskenya.org/

[28] International Diabetes Federation . *IDF Diabetes Atlas*. 10th ed. International Diabetes Federation; 2021.

[29] FIND . Improving Access to Continuous Glucose Monitoring Devices in Kenya and South Africa. Accessed April 2024, 2024. https://www.finddx.org/what-we-do/projects/improving-access-to-continuous-glucose-monitoring-devices-in-kenya-and-south-africa/

[30] Sifunda S, Mbewu AD, Mabaso M, et al Prevalence and psychosocial correlates of diabetes mellitus in South Africa: results from the South African national health and nutrition examination survey (SANHANES-1). Int J Environ Res Public Health. 2023;20(10):5798. 37239526 10.3390/ijerph20105798 PMC10218408

[31] Australian Bureau of Statistics. Diabetes . ABS. Accessed May 22, 2024. https://www.abs.gov.au/statistics/health/health-conditions-and-risks/diabetes/latest-release

[32] Lomax KE, Taplin CE, Abraham MB, et al Socioeconomic status and diabetes technology use in youth with type 1 diabetes: a comparison of two funding models. Front Endocrinol (Lausanne). 2023;14:1178958. 37670884 10.3389/fendo.2023.1178958 PMC10476216

[33] Understanding Type 1 Diabetes . Diabetes New Zealand. Accessed September 18, 2024. https://www.diabetes.org.nz/type1diabetes

[34] World Health Organization . Switzerland: Health data overview for the Swiss Confederation. World Health Organization. Accessed September 18, 2024. https://data.who.int/countries/756

[35] Addala A, Auzanneau M, Miller K, et al A decade of disparities in diabetes technology use and HbA(1c) in pediatric type 1 diabetes: a transatlantic comparison. Diabetes Care. 2021;44(1):133‐140. 32938745 10.2337/dc20-0257 PMC8162452

[36] Lorenzen JT, Madsen KP, Cleal B, et al Associations between use of diabetes technology and diabetes distress: a Danish cross-sectional survey of adults with type 1 diabetes. BMJ Open. 2024;14(3):e080053. 10.1136/bmjopen-2023-080053 PMC10966817 38531585

[37] Bratke H, Biringer E, Margeirsdottir HD, Njølstad PR, Skrivarhaug T. Relation of health-related quality of life with glycemic control and use of diabetes technology in children and adolescents with type 1 diabetes: results from a national population based study. J Diabetes Res. 2022;2022:8401328. 36387938 10.1155/2022/8401328 PMC9649325

[38] Wersäll JH, Adolfsson P, Forsander G, Hanas R. Insulin pump therapy is associated with higher rates of mild diabetic ketoacidosis compared to injection therapy: a 2-year Swedish national survey of children and adolescents with type 1 diabetes. Pediatr Diabetes. 2022;23(7):1038‐1044. 35678764 10.1111/pedi.13377 PMC9796597

[39] Svensson A-M, Eliasson B, Linder E, Almskog I, Hermansson-Carter V, Eeg-Olofsson K. *Nationwide Results 1996-2019*. Swedish National Diabetes Register NDR. Doi: 10.18158/ryNUNVPiu

[40] Lee MY, Tanenbaum ML, Maahs DM, Prahalad P. Overcoming barriers to diabetes technology in youth with type 1 diabetes and public insurance: cases and call to action. Case Rep Endocrinol. 2022;2022:9911736. 35273814 10.1155/2022/9911736 PMC8904094

[41] Tanenbaum ML, Hanes SJ, Miller KM, Naranjo D, Bensen R, Hood KK. Diabetes device use in adults with type 1 diabetes: barriers to uptake and potential intervention targets. Diabetes Care. 2017;40(2):181‐187. 27899489 10.2337/dc16-1536 PMC5864141

[42] Walker AF, Hood KK, Gurka MJ, et al Barriers to technology use and endocrinology care for underserved communities with type 1 diabetes. Diabetes Care. 2021;44(7):1480‐1490. 34001535 10.2337/dc20-2753 PMC8323174

[43] Yamamoto Y, Kikuchi T, Urakami T, et al Status and trends in the use of insulin analogs, insulin delivery systems and their association with glycemic control: comparison of the two consecutive recent cohorts of Japanese children and adolescents with type 1 diabetes mellitus. J Pediatr Endocrinol Metab. 2019;32(1):1‐9. 30517078 10.1515/jpem-2018-0329

[44] Shimmura S, Kawamura T. Problems with real-time CGM and CSII (in Japanese). Curr Treat Diabetes. 2018;9(4):188‐191.

[45] Yilan County Love Pancreas Association Home page . August 19, 2024, 2024. Taiwan. Accessed August 19, 2024. https://www.dmsupport.org.tw/

[46] Ong WM, Chua SS, Ng CJ. Barriers and facilitators to self-monitoring of blood glucose in people with type 2 diabetes using insulin: a qualitative study. Patient Prefer Adherence. 2014;8:237‐246. 24627628 10.2147/PPA.S57567 PMC3931581

[47] Burnside MJ, Williman JA, Davies HM, et al Inequity in access to continuous glucose monitoring and health outcomes in paediatric diabetes, a case for national continuous glucose monitoring funding: a cross-sectional population study of children with type 1 diabetes in New Zealand. Lancet Reg Health West Pac. 2023;31:100644. 36419466 10.1016/j.lanwpc.2022.100644 PMC9676142

[48] Rotondi MA, Wong O, Riddell M, Perkins B. Population-level impact and cost-effectiveness of continuous glucose monitoring and intermittently scanned continuous glucose monitoring technologies for adults with type 1 diabetes in Canada: a modeling study. Diabetes Care. 2022;45(9):2012‐2019. 35834175 10.2337/dc21-2341 PMC9472499

[49] Diabetes Canada Clinical Practice Guidelines Expert Working Group; Cheng AYY, Feig DS, et al Blood glucose monitoring in adults and children with diabetes: update 2021. Can J Diabetes. 2021;45(7):580‐587. 34511234 10.1016/j.jcjd.2021.07.003

[50] Centers for Disease Control and Prevention . *National Diabetes Statistics Report*. 2024. Accessed May 15, 2024. https://www.cdc.gov/diabetes/php/data-research/

[51] Conway RB, Gerard Gonzalez A, Shah VN, et al Racial disparities in diabetes technology adoption and their association with HbA1c and diabetic ketoacidosis. Diabetes Metab Syndr Obes. 2023;16:2295‐2310. 37551339 10.2147/DMSO.S416192 PMC10404403

[52] Lin MH, Connor CG, Ruedy KJ, et al Race, socioeconomic status, and treatment center are associated with insulin pump therapy in youth in the first year following diagnosis of type 1 diabetes. Diabetes Technol Ther. 2013;15(11):929‐934. 23869706 10.1089/dia.2013.0132 PMC3817890

[53] Paris CA, Imperatore G, Klingensmith G, et al Predictors of insulin regimens and impact on outcomes in youth with type 1 diabetes: the SEARCH for diabetes in youth study. J Pediatr. 2009;155(2):183‐189.e1. 19394043 10.1016/j.jpeds.2009.01.063

[54] Snyder LL, Stafford JM, Dabelea D, et al Socio-economic, demographic, and clinical correlates of poor glycaemic control within insulin regimens among children with type 1 diabetes: the SEARCH for diabetes in youth study. Diabet Med. 2019;36(8):1028‐1036. 31050009 10.1111/dme.13983 PMC6635011

[55] Grassi B, Gómez AM, Calliari LE, et al Real-world performance of the MiniMed 780G advanced hybrid closed loop system in Latin America: substantial improvement in glycaemic control with each technology iteration of the MiniMed automated insulin delivery system. Diabetes Obes Metab. 2023;25(6):1688‐1697. 36789699 10.1111/dom.15023

[56] Antonio-Villa NE, García-Tuomola A, Almeda-Valdes P, et al Glycemic control, treatment and complications in patients with type 1 diabetes amongst healthcare settings in Mexico. Diabetes Res Clin Pract. 2021;180:109038. 34487758 10.1016/j.diabres.2021.109038

[57] Patel JV, Alexander M, Flinders P. Diabetes mellitus: the Latin American paradox. Int J Clin Pract. 2013;67(12):1217‐1218. 24246201 10.1111/ijcp.12257

[58] Borges LP, de Jesus PC, de Souza JB, et al The impact of diabetes education on continuous glucose monitoring in SUS-dependent patients in a northeastern Brazilian city. Life (Basel). 2024;14(3). Doi: 10.3390/life14030320 PMC10971600 38541647

[59] Bahia L, Mello KF, Lemos LLP, Costa NL, Mulinari E, Malerbi DA. Cost-effectiveness of continuous glucose monitoring with FreeStyle Libre® in Brazilian insulin-treated patients with types 1 and 2 diabetes mellitus. Diabetol Metab Syndr. 2023;15(1):242. 38001509 10.1186/s13098-023-01208-5 PMC10675900

[60] Green A, Hede SM, Patterson CC, et al Type 1 diabetes in 2017: global estimates of incident and prevalent cases in children and adults. Diabetologia. 2021;64(12):2741‐2750. 34599655 10.1007/s00125-021-05571-8 PMC8563635

[61] Kawasaki E, Matsuura N, Eguchi K. Type 1 diabetes in Japan. Diabetologia. 2006;49(5):828‐836. 16568259 10.1007/s00125-006-0213-8

[62] Onda Y, Sugihara S, Ogata T, Yokoya S, Yokoyama T, Tajima N. Incidence and prevalence of childhood-onset type 1 diabetes in Japan: the T1D study. Diabet Med. 2017;34(7):909‐915. 27925270 10.1111/dme.13295

[63] Matsuda F, Itonaga T, Maeda M, Ihara K. Long-term trends of pediatric type 1 diabetes incidence in Japan before and after the COVID-19 pandemic. Sci Rep. 2023;13(1):5803. 37037893 10.1038/s41598-023-33037-x PMC10085994

[64] Lee YB, Kim M, Kim JH. Glycemia according to the use of continuous glucose monitoring among adults with type 1 diabetes mellitus in Korea: a real-world study. Diabetes Metab J. 2023;47(3):405‐414. 36872066 10.4093/dmj.2022.0032 PMC10244200

[65] Lu CL, Chang YH, Martini S, Chang MF, Li CY. Overall and cause-specific mortality in patients with type 1 diabetes mellitus: a population-based cohort study in Taiwan from 1998 through 2014. J Epidemiol. 2021;31(9):503‐510. 32741854 10.2188/jea.JE20200026 PMC8328860

[66] Hussein Z, Taher SW, Gilcharan Singh HK, Chee Siew Swee W. Diabetes care in Malaysia: problems, new models, and solutions. Ann Glob Health. 2018;81(6):851‐862. 10.1016/j.aogh.2015.12.016 27108152

[67] Management of Type 1 Diabetes Mellitus in Children & Adolescents. Malaysia Health Technology Assessment Section (MaHTAS); 2015.

[68] Fralick M, Jenkins AJ, Khunti K, Mbanya JC, Mohan V, Schmidt MI. Global accessibility of therapeutics for diabetes mellitus. Nat Rev Endocrinol. 2022;18(4):199‐204. 35039662 10.1038/s41574-021-00621-y PMC8762447

[69] Kiran S, Nagarajappa VH, Sathyanarayana SO, Hegde A, Raghupathy P. Use of continuous glucose monitoring system in children with type 1 diabetes mellitus in a resource limited setting. Indian J Endocrinol Metab. 2023;27(3):208‐212. 37583401 10.4103/ijem.ijem_376_22 PMC10424105

[70] Swaminathan K . One-year follow-up of 50 rural underprivileged type 1 diabetes children on insulin pump therapy: breaking socio-economic barriers in diabetes technologies. Indian J Endocrinol Metab. 2023;27(3):213‐215. 37583411 10.4103/ijem.ijem_324_22 PMC10424104

[71] Mandaviya M, Pawar BP. Guidelines for Management of Type 1 Diabetes. Indian Council of Medical Research; 2022.

[72] Atun R, Davies JI, Gale EAM, et al Diabetes in sub-Saharan Africa: from clinical care to health policy. Lancet Diabetes Endocrinol. 2017;5(8):622‐667. 28688818 10.1016/S2213-8587(17)30181-X

[73] Beran D, Lazo-Porras M, Mba CM, Mbanya JC. A global perspective on the issue of access to insulin. Diabetologia. 2021;64(5):954‐962. 33483763 10.1007/s00125-020-05375-2 PMC8012321

[74] Beran D, Laing RO, Kaplan W, et al A perspective on global access to insulin: a descriptive study of the market, trade flows and prices. Diabet Med. 2019;36(6):726‐733. 30888075 10.1111/dme.13947 PMC6593686

[75] Virmani A, Brink SJ, Middlehurst A, et al ISPAD clinical practice consensus guidelines 2022: management of the child, adolescent, and young adult with diabetes in limited resource settings. Pediatr Diabetes. 2022;23(8):1529‐1551. 36537524 10.1111/pedi.13456

[76] Lomax KE, Taplin CE, Abraham MB, et al Improved glycemic outcomes with diabetes technology use independent of socioeconomic status in youth with type 1 diabetes. Diabetes Care. 2024;47(4):707‐711. 38324670 10.2337/dc23-2033

[77] Karges B, Schwandt A, Heidtmann B, et al Association of insulin pump therapy vs insulin injection therapy with severe hypoglycemia, ketoacidosis, and glycemic control among children, adolescents, and young adults with type 1 diabetes. JAMA. 2017;318(14):1358‐1366. 29049584 10.1001/jama.2017.13994 PMC5818842

[78] Kordonouri O, Lange K, Biester T, et al Determinants of glycaemic outcome in the current practice of care for young people up to 21 years old with type 1 diabetes under real-life conditions. Diabet Med. 2020;37(5):797‐804. 31498923 10.1111/dme.14130

[79] DeSalvo DJ, Lanzinger S, Noor N, et al Transatlantic comparison of pediatric continuous glucose monitoring use in the diabetes-patienten-verlaufsdokumentation initiative and type 1 diabetes exchange quality improvement collaborative. Diabetes Technol Ther. 2022;24(12):920‐924. 35947079 10.1089/dia.2022.0248

[80] Auzanneau M, Eckert AJ, Meyhöfer SM, et al Area deprivation and demographic factors associated with diabetes technology use in adults with type 1 diabetes in Germany. Front Endocrinol (Lausanne). 2023;14:1191138. 37600703 10.3389/fendo.2023.1191138 PMC10433185

[81] Dennig MJ, Sommer G, Zingg T, Flück CE, Boettcher C. Stable metabolic control but increased demand for professional support in children with type 1 diabetes in the past ten years in Bern/Switzerland: a quality control study. J Diabetes Res. 2022;2022:3170558. 36034586 10.1155/2022/3170558 PMC9402297

[82] Nathanson D, Svensson AM, Miftaraj M, Franzén S, Bolinder J, Eeg-Olofsson K. Effect of flash glucose monitoring in adults with type 1 diabetes: a nationwide, longitudinal observational study of 14,372 flash users compared with 7691 glucose sensor naive controls. Diabetologia. 2021;64(7):1595‐1603. 33774713 10.1007/s00125-021-05437-z PMC8187189

[83] Europe IDF . *Insulin at 100. An Overview of Diabetes Care: Norway*. Accessed May 10, 2024. https://www.insulin100.eu/wp-content/uploads/2023/04/Country-Profile-Norway-003.pdf

[84] Kubiak T, Priesterroth L, Barnard-Kelly KD. Psychosocial aspects of diabetes technology. Diabet Med. 2020;37(3):448‐454. 31943354 10.1111/dme.14234

[85] Tanenbaum ML, Adams RN, Hanes SJ, et al Optimal use of diabetes devices: clinician perspectives on barriers and adherence to device use. J Diabetes Sci Technol. 2017;11(3):484‐492. 28745093 10.1177/1932296816688010 PMC5505431

[86] Kubiak T, Mann CG, Barnard KC, Heinemann L. Psychosocial aspects of continuous glucose monitoring: connecting to the patients’ experience. J Diabetes Sci Technol. 2016;10(4):859‐863. 27234808 10.1177/1932296816651450 PMC4928243

[87] Borges U, Kubiak T. Continuous glucose monitoring in type 1 diabetes. J Diabetes Sci Technol. 2016;10(3):633‐639. 26961974 10.1177/1932296816634736 PMC5038544

[88] Vloemans AF, van Beers CAJ, de Wit M, et al Keeping safe. Continuous glucose monitoring (CGM) in persons with type 1 diabetes and impaired awareness of hypoglycaemia: a qualitative study. Diabet Med. 2017;34(10):1470‐1476. 28731509 10.1111/dme.13429

[89] Ogle GD, von Oettingen JE, Middlehurst AC, Hanas R, Orchard TJ. Levels of type 1 diabetes care in children and adolescents for countries at varying resource levels. Pediatr Diabetes. 2019;20(1):93‐98. 30471084 10.1111/pedi.12801

