# Correction to: “Disparities in Diabetes Technology Uptake in Youth and Young Adults With Type 1 Diabetes: A Global Perspective”

**DOI:** 10.1210/jendso/bvaf071

**Published:** 2025-05-09

In the above-named article by Conway RB, Snell-Bergeon J, Honda-Kohmo K, Peddi AK, Isa SB, Sulong S, Sibomana L, Gonzalez AG, Song J, Lomax KE, Lo C-N, Kim W, Haynes A, de Bock M, Burckhardt M-A, Schwab S, and Hong K (*J Endo Society.* 2025; 9(1); doi: 10.1210/jendso/bvae210), there was an error in the Methods section.

In the originally published article, in the Methods section, the URL “http://medicalonline.jp” was provided with a missing “s” in the beginning of the web address. In the corrected article, the URL has been updated to “https://www.medicalonline.jp.”

The article has been corrected online.

doi: 10.1210/jendso/bvae210

